# Enhancing the Efficacy of Chiral Ligands and Catalysts: Siloxane-Substituted Oxazoline Ferrocenes as Next-Generation Candidates

**DOI:** 10.3390/molecules29050968

**Published:** 2024-02-22

**Authors:** Li Dai, Li Zhao, Di Xu, Chen Yang, Xin-Kuan Zhang

**Affiliations:** 1 School of Environmental and Municipal Engineering, North China University of Water Resources and Electric Power, Zhengzhou 450046, China; daili@ncwu.edu.cn (L.D.); 201923625@stu.ncwu.edu.cn (L.Z.); 202012607@stu.ncwu.edu.cn (X.-K.Z.); 2 Collaborative Innovation Center for Efficient Utilization of Water Resources, North China University of Water Resources and Electric Power, Zhengzhou 450046, China; yangchen@ncwu.edu.cn

**Keywords:** oxazoline, ferrocene, planar chirality, chiral ligands, catalyst recycling

## Abstract

Since the discovery of classical chiral oxazoline ferrocene ligands in 1995, they have become pivotal in transition metal-catalyzed asymmetric transformations. Over the past decade, a notable evolution has been observed with the emergence of siloxane-substituted oxazoline ferrocenes, demonstrating significant potential as chiral ligands and catalysts. These compounds have consistently delivered exceptional results in diverse and mechanistically distinct transformations, surpassing the capabilities of classical oxazoline ferrocene ligands. This review meticulously delineates the research progress on siloxane-substituted oxazoline ferrocene compounds. It encompasses the synthesis of crucial precursors and desired products, highlights their achievements in asymmetric catalysis reactions, and delves into the exploration of the derivatization of these compounds, emphasizing the introduction of ionophilic groups and their impact on the recovery of transition metal catalysts. In addition to presenting the current state of knowledge, this review propels future research directions by identifying potential topics for further investigation concerning the siloxane-tagged derivatives. These derivatives are poised to be promising candidates for the next generation of highly efficient ligands and catalysts.

## 1. Introduction

Ferrocene-type compounds have captivated the attention of organic chemists as versatile chiral ligands and catalysts. The appeal lies not only in the ease of preparing poly-substituted ferrocene compounds through electrophilic substitutions, leveraging the higher electron density in the cyclopentadienyl (*Cp*) ring compared to benzene, but also in the creation of “planar chirality” by introducing multiple substituents, breaking the symmetry plane of ferrocene. This distinctive feature enhances the efficacy of ferrocene-based ligands and catalysts in asymmetric catalysis transformations. Originating from independent research in 1995 by Richards [1], Sammakia [2], and Uemura’s [3] groups, chiral ligands based on the oxazoline ferrocene backbone have played a pivotal role in diverse transition metal-catalyzed asymmetric reactions over the past three decades, ranging from academic laboratory researches to industrial productions [4,5,6], with more than 600 publications and patents attesting to their significance. Even today, chiral oxazoline phosphine ferrocene ligands (*FOXAP*s) remain the preferred choice for many emerging reactions aimed at achieving highly stereoselective products [7,8,9,10,11,12,13,14,15,16,17,18,19,20,21,22].

**Figure 1:**
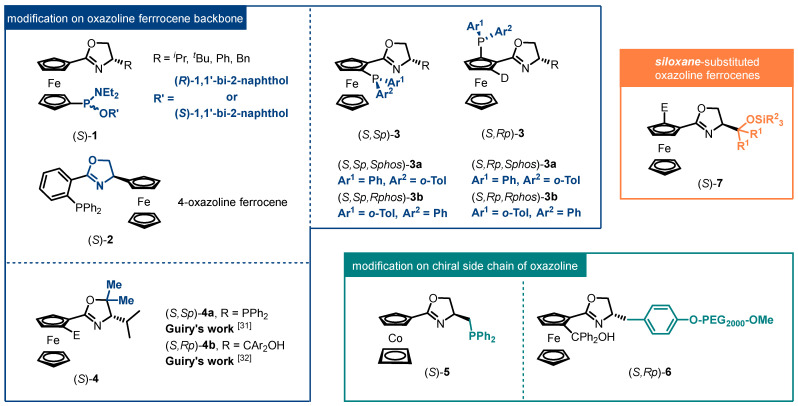
Modified ligands from oxazoline metallocene skeleton.

With the continued research of oxazoline ferrocene ligands, research on their structural modification has also been ongoing (Figure 1). The *first* strategy is the exploration of substituent patterns. For instance, Hou developed a class of 1,1′-*N*,*P* bidentate ligands **1** from 2001 [23]. The applications of these ligands have demonstrated that the established ligands provide powerful stereo control on the corresponding chiral products in the aggregation of Pd-catalyzed asymmetric reactions [24,25,26]. Alternatively, in 2002, based on the seminal work from Patti’s group [27], Moyano and co-workers delivered the absolute configuration of chiral 4-oxazoline ferrocene **2**, and then examined the applicability in asymmetric allylic alkylation of *rac*-(*E*)-1,3-diphenyl-2-propenyl acetate, which provided the target alkylated product with 97:3 e.r. [28]. The *second* strategy is the exploration of donor groups. Several donor groups have been introduced into the oxazoline ferrocene backbone to establish their corresponding chiral ligands, which are utilized in various asymmetric mechanistically different transformations. Notably, Richards recently proposed a novel synthetic scheme for FOXAP ligands **3**, which incorporates three chiral elements [29]. The strategy involves the use of a “deuterium blocking group” to control the absolute configuration of planar chirality [30] and a stepwise stereoselective synthesis of *P*-stereogenic diastereoisomers. The catalytic results revealed that the phosphorus-based stereogenic center had a positive influence on the enantioselectivity of the alkylated products in the presence of matched elements of chirality. The *third* strategy is the exploration of oxazoline ferrocene backbone modification. An example of structural modification was presented by Guiry, who reported the synthesis of a series of novel chiral 5,5-*gem*-disubstituted oxazoline ferrocene ligands **4** [31,32]. The Zn-mediated enantioselective nucleophilic additions to aldehydes with the generated ligands provided a wide range of secondary alcohols with yields up to 93% and up to over 99% *ee*.

In comparison, there are limited studies on the modification of chiral substituents in oxazoline moieties. The chiral side chains in oxazoline are derived from simple chiral amino alcohols. As a result, traditional chiral substituents in oxazoline ferrocene ligands are alkyl groups (such as *iso*-propyl, *tert*-butyl, etc.) and aryl groups (such as phenyl and benzyl). Functionalized substituents usually imply relatively complicated synthesis processes and specialized starting materials. Richards’ group, who devoted their efforts on the research on mono-substituted oxazoline metallocene ligands **5**, established a straightforward five steps for chiral *N*,*P*-ligand from (*S*)-Serine methyl ester [33,34]. In 2016, Liu reported the preparation scheme of desired uncommon chiral amino alcohol from commercial available *L*-tyrosine in five continuous reactions, which was designed as a crucial substrate for MeO-PEG-supported chiral oxazoline carbinol ferrocene ligand **6** [35].

In particular, the design of the side chain of the oxazoline ring has been identified as a crucial aspect in achieving highly efficient catalysts. The steric bulkiness generated by chiral oxazoline side chains imparts planar chiral ferrocene derivatives with high diastereoselectivity and rigid hindrance for prochiral substrates. This property has proven instrumental in directed *ortho*-metallation processes and transition metal-catalyzed asymmetric reactions, resulting in satisfactory stereoselective outcomes. Amidst this context, tunable siloxane-substituted oxazoline ferrocenes **7** have emerged as promising candidates for the next generation of highly efficient ligands and catalysts. Their tunability and potential for further derivatization offer exciting possibilities for applications in emerging reactions requiring precise steric resistance. In the last decade, tunable siloxane-substituted oxazoline ferrocenes have demonstrated impressive results in various transition metal-catalyzed asymmetric transformations, solidifying their potential in advancing catalytic methodologies.

While critical reviews on chiral oxazoline ferrocene ligands exist, most focus on classical ligands with traditional chiral substituents [36,37,38,39,40]. This brief review seeks to consolidate the knowledge on chiral siloxane-substituted oxazoline ferrocenes, shedding light on their achievements in asymmetric reactions and paving the way for potential developments in this specialized class of compounds.

## 2. Synthetic Strategies of Siloxane-Substituted Oxazoline Ferrocene Ligands

### 2.1. Enantiopure Siloxane-Substituted Oxazoline Ferrocenes

The development of efficient methods to obtain optically pure oxazoline ferrocene compounds is crucial for achieving highly diastereoselective planar chiral ligands through direct *ortho*-metallation of ferrocene. In our recent review, we comprehensively summarized the synthesis methods for 2-oxazoline ferrocene derivatives [40]. Currently, three independent strategies for synthesizing siloxane-substituted oxazoline ferrocenes are discussed in this article, illustrated in the following reaction schemes.

**Scheme 1:**
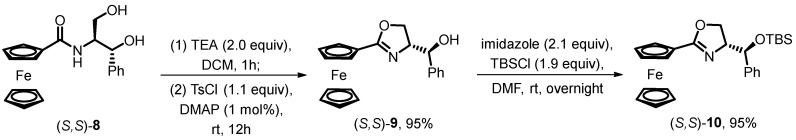
Synthesis of siloxane-substituted oxazoline ferrocene by Aït-Haddou’s group.

In 2000, Aït-Haddou and Balavoine’s group reported a facile cyclization method for oxazoline ferrocene on a large scale (Scheme 1) [41]. The starting material, chiral *β*-hydroxyl ferrocene amide **8**, is prepared by the condensation of ferrocenyl carbonyl chloride with a slight excess of (1*S*,2*S*)-2-amino-3-phenyl-1,3-dipropanediol. It is then treated with TsCl and a catalytic amount of DMAP to yield the oxazoline ferrocene analogue **9** in an excellent yield of 95%. Before investigating the effectiveness of the oxazoline fragment in diastereoselective *ortho*-lithiation, the free hydroxyl group on the side chain of the oxazoline unit is protected by TBSCl in DMF, resulting in the desired siloxane-substituted oxazoline ferrocene **10** with a yield of 95%.

**Scheme 2:**
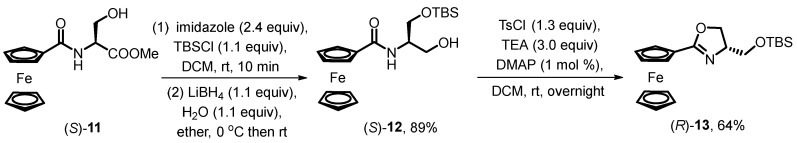
Synthesis of siloxane-substituted oxazoline ferrocene by Sammakia’s group.

Sammakia, recognized as one of the pioneers in the application of chiral oxazoline-substituted ferrocene-type ligands, proposed an alternative route for silyl-protected oxazoline ferrocene (Scheme 2) [2]. Chiral *β*-hydroxyl ferrocene amides **11** are prepared through the condensation of ferrocenyl carbonyl chloride and (*S*)-serine methyl ester hydrochloride. Subsequent silylation of the hydroxyl group of **12** by TBSCl precedes the reduction procedure of the ester group to avoid the formation of an achiral cyclization product. The desired silyl-protected oxazoline ferrocene **13** is then prepared from **12** in a ring-closure process by the treatment of TsCl and trimethylamine, yielding 64% after flash chromatography purification.

**Scheme 3:**
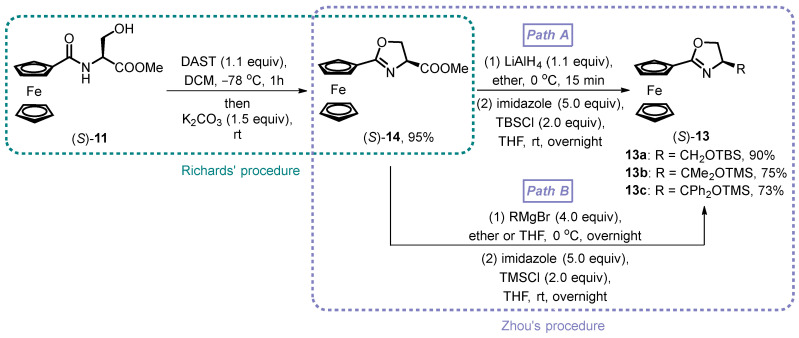
Synthesis of siloxane-substituted oxazoline ferrocene by Zhou’s procedure.

Richards and co-workers presented a direct process for chiral oxazoline ring formation (Scheme 3) [33,34]. The establishment of oxazoline metallocenes **14** is performed from the serine-derived *β*-hydroxy amides **11** using 1.1 equivalents of DAST at −78 °C for 1 h, followed by the addition of anhydrous K_2_CO_3_, resulting in the corresponding products in almost quantitative yield. Subsequently, Zhou reported a synthetic route extension of **14** with a reduction of the carboxylic ester group by LiAlH_4_ in ether and a subsequent silylation of the produced hydroxyl group with TBSCl in anhydrous THF. The target siloxane-substituted oxazoline ferrocene (*S*)-**13**, outlined as an enantiomeric isomer in Sammakia’s work, is finally achieved as a red viscous liquid in 90% yield in two steps (Scheme 3, Path A) [42]. Moreover, the chiral side chain of the oxazoline fragment was then extended by Zhou’s subsequent work as **13b** and **13c** with good conversion (Scheme 3, Path B).

### 2.2. Synthesis of Planar Chiral Oxazoline Ferrocene Derivatives

Planar chiral 1,2-disubstituted ferrocene-type compounds stand out as the most preferred ligands in research focused on their applications in asymmetric transformations. Consequently, the preparation schemes of these planar chiral ferrocenes, which yield highly stereochemical outcomes, have garnered attention from various organic chemistry research groups. Chiral oxazoline groups have proven to be accessible directing groups for establishing 1,2-disubstituted ferrocenes through a diastereoselective direct *ortho*-metallation (D*o*M) procedure. Sammakia reported a dramatic diastereoselectivity of planar chiral products, achieving an over 500:1 diastereoisomer ratio upon the addition of *N*,*N*,*N*′,*N*′-tetramethylethylenediamine (TMEDA) [43]. The D*o*M procedure with chiral oxazoline moieties has been theoretically supported by several experimental and computational results [44,45,46]. However, in comparison to conventional chiral oxazoline ferrocenes, the D*o*M procedures with enantiopure siloxane-substituted oxazoline ferrocenes as starting materials remain challenging but intriguing topics in current research.

#### 2.2.1. Sulfur and Phosphine Ligands

**Scheme 4:**
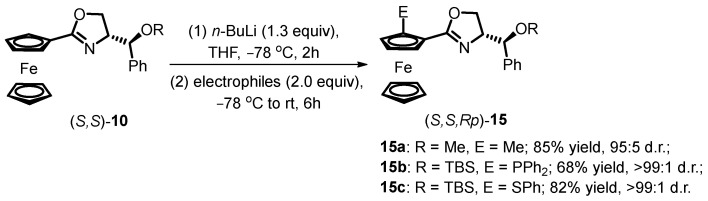
D*o*M procedures of oxazoline ferrocene with two stereogenic centers.

Aït-Haddou and colleagues investigated the possibility of diastereoselective *ortho*-functionalization using oxazoline ferrocenes as substrates, bearing two stereogenic centers [41]. The deprotonation reaction was conducted with 1.3 equivalents of RLi at −78 °C in THF for 2 h, followed by the addition of selected electrophiles (Scheme 4). Results demonstrated high diastereoselectivities exceeding 95:5 d.r. of **15a** in the absence of additional additives, while OMe group was attached to the side chain of oxazoline. Notably, when utilizing siloxane-substituted oxazoline ferrocene derivatives, the diastereoselectivity of **15b** increased to 99:1 due to enhanced steric hindrance established by the siloxane moiety. The researchers concluded that *N*,*S*- and *N*,*P*-ligands could be generated using the same procedure by varying the electrophilic reagents, offering versatility in subsequent asymmetric reactions.

**Scheme 5:**
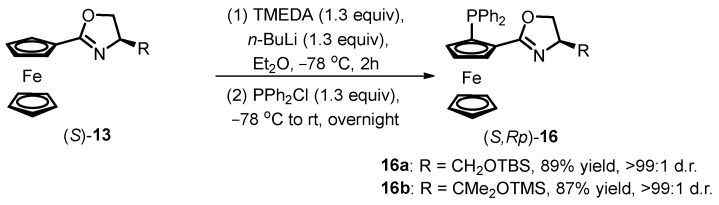
D*o*M procedures of oxazoline ferrocenes with the addition of TMEDA.

Zhou explored the facile preparation of planar chiral siloxane-substituted oxazoline phosphine ferrocene ligand from the readily generated ferrocene compounds through a standard diastereoselective direct *ortho*-metallation (D*o*M) procedure [42]. Enantiopure (*S*)-**13** was treated with a slight excess of *n*-BuLi under −78 °C with the addition of TMEDA in ether for 2 h. The preferred electrophile PPh_2_Cl was then added, and the mixture was allowed to stir overnight. The designed ligand (*S*,*Rp*)-**16a** was obtained in 89% yield after purification through column chromatography. In their subsequent work, the identical method was employed to synthesize ligand (*S*,*Rp*)-**16b**, introducing a larger steric hindrance on the chiral side chain of the oxazoline fragment (Scheme 5) [47].

However, recent research has unveiled a decrease in diastereoselectivity during the D*o*M process when a bulky substituent is present on the oxazoline chiral side chain. In the treatment of siloxane-substituted oxazoline ferrocene (*S*)-**13c**, the conversion of (*S*,*Rp*)-**16c** was regarded as only 75%, and a significant reduction in diastereoselectivity was observed (d.r. = 4.5:1, Scheme 6). The hypothesis put forward suggests that the excessively bulky substituent may decelerate the lithiation reaction rate and diminish regioselectivity during the lithiation process.

#### 2.2.2. Bisphosphine Ligands

**Scheme 7:**
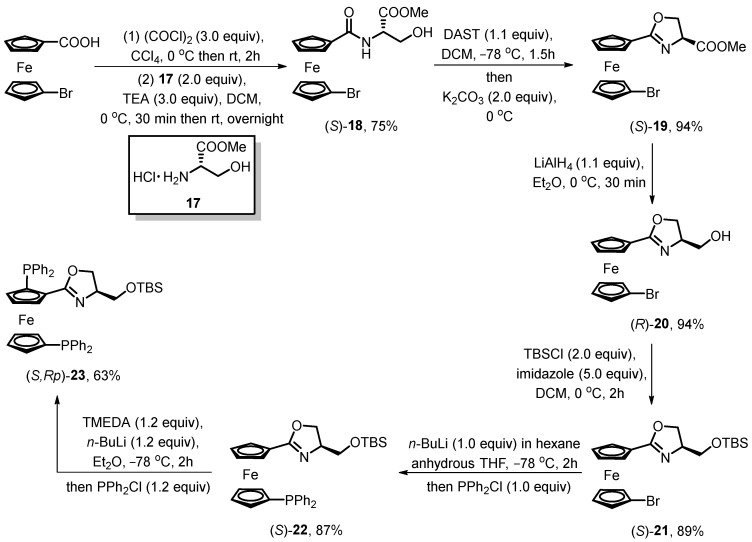
Synthetic approach of chiral oxazoline 1,2′-bisphosphine ferrocene.

In 2019, Dai reported groundbreaking work on the chiral bisphosphine oxazoline ferrocene derivative with a siloxane substituent group [48]. In contrast to the synthesis method of mono-phosphine ligands **16**, this study successfully synthesized the 1,2′-bisphosphine ferrocene compound using 1-bromo-1′-carboxyferrocene as the starting material (Scheme 7). The oxazoline ring formation followed Richards’ synthesis process, with the hydroxyl ferrocene amide (*S*)-**18** participating in the cyclization reaction under the treatment of DAST. The side-chain modification of the oxazoline moiety adopted Zhou’s scheme, leading to the successful synthesis of siloxane-substituted oxazoline ferrocene (*S*)-**19**. Prior to the directed *ortho*-lithiation process, a phosphonation reaction was carried out on the lower *Cp* ring at the preset bromine atom, resulting in the formation of 1,1′-*N*,*P*-oxazoline ferrocene derivative (*S*)-**22**. The analytically pure oxazoline bisphosphine ferrocene (*S*,*Rp*)-**23** was finally generated in six steps with an overall yield of 32% from the starting material. The presented bisphosphine ferrocene compound is considered a potential ligand for transition metal-catalyzed asymmetric allylic alkylation and Heck reactions.

**Scheme 8:**
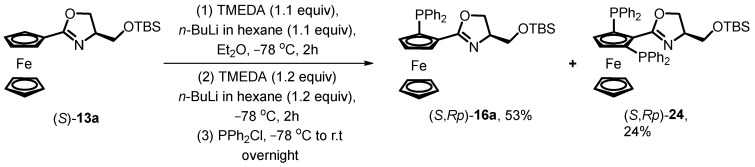
Stepwise lithiation of chiral oxazoline ferrocene.

The authors also explored the possibility of obtaining the desired product from enantiopure oxazoline ferrocene (*S*)-**13a** through a stepwise lithiation process reported by Kumada for the establishment of 1,2′-bisphosphine ferrocene ligands BPPFA from Ugi’s amine [49]. However, under the standard stepwise lithiation process conditions, the appearance of the 2,5-bisphosphine product (*S*,*Rp*)-**24** demonstrated the failure to obtain the desired product due to the possible simultaneous *N*-directed and *O*-directed *ortho*-lithiation induced by oxazoline as a chiral auxiliary (Scheme 8).

#### 2.2.3. Hydroxyl Ligands (Catalysts)

**Figure 2:**
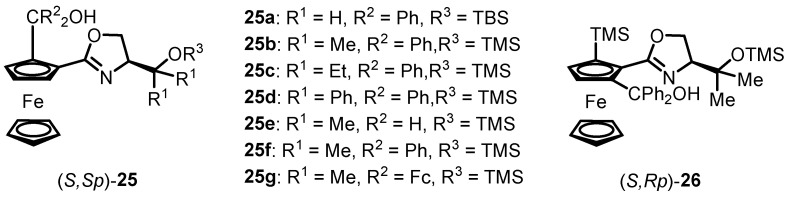
Structures of siloxane-substituted hydroxyl oxazoline ferrocenes.

Our research group has recently presented an innovative synthesis scheme for siloxane-substituted hydroxyl oxazoline ferrocene compounds [50]. Classical hydroxyl oxazoline ferrocene compounds are widely employed as chiral ligands, demonstrating excellent results in various Zn-catalyzed enantioselective nucleophilic additions to aldehydes [39]. Catalytic findings have revealed that the chiral side-chain structure of oxazoline ferrocene, the type of coordinative carbinol groups, and the absolute planar chiral configuration of the participating ligands play crucial roles in determining the stereoselectivity of the optically active products. In this research report, the structure of siloxane-substituted ligands has been effectively tailored, leading to the preparation of ligands (*S*,*Sp*)-**25** and (*S*,*Rp*)-**26** with distinct chiral side-chain substituents, carbinol groups, and planar chirality, as listed in Figure 2.

**Scheme 9:**
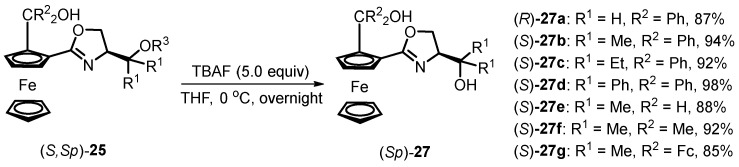
Praparation of *N*,*O*,*O*-tridentate ferrocenes via a desilylation reaction. The structure of R_3_ in (*S*,*Sp*)-**25** is presented in Figure 2.

Notably, a novel series of *N*,*O*,*O*-tridentate ligands can be easily synthesized from chiral oxazolinyl hydroxyl ferrocene ligands (*S*,*Sp*)-**25** with siloxane substitution (Scheme 9). The desilylation reaction was executed by reacting the synthesized siloxane-substituted oxazoline ferrocene compounds with 5.0 equivalents of TBAF in THF at 0 °C under an inert atmosphere. The desired desilylated compounds (*Sp*)-**27** were obtained in high yield for most products. Our laboratory is currently exploring the application of siloxane-substituted *N*,*O*-ligands **25** and **26** and their corresponding desilylated *N*,*O*,*O*-ligands **27** in enantioselective nucleophilic additions and alkynylation to aldehydes.

## 3. Applications of Siloxane-Substituted Oxazoline Ferrocene Ligand in Transition Metal-Catalyzed Reactions

### 3.1. Pd-Catalyzed Asymmetric Allylic Alkylation

**Scheme 10:**
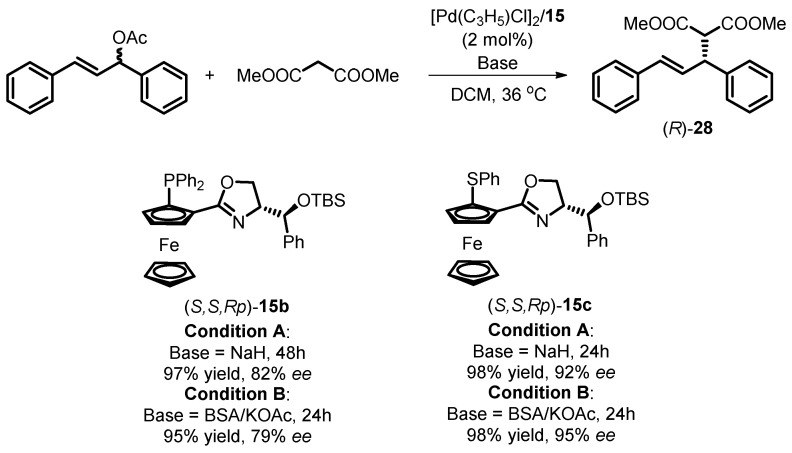
Palladium-catalyzed asymmetric allylic alkylation with siloxane-substituted oxazoline ferrocene ligands.

Aït-Haddou and colleagues employed the synthesized bidentate oxazoline ferrocene compounds (*S*,*S*,*Rp*)-**15** as chiral ligands in a palladium-catalyzed allylic alkylation reaction [41]. This reaction involved the use of *rac*-1,3-diphenylprop-2-enyl acetate as the substrate and dimethyl malonate as the nucleophile (Scheme 10). The Pd complex catalysts were generated in situ from the readily prepared ligands and bis[(*η*-allyl)palladium chloride]. The asymmetric allylic substitution took place smoothly in dichloromethane at 36 °C using this Pd complex catalyst. Notably, when employing *S*,*N*-bidentate oxazoline ferrocene ligand (*S*,*S*,*Rp*)-**15c**, the catalytic reaction yielded alkylation products with high enantioselectivity and almost quantitative yields. This held true regardless of whether NaH or BSA/KOAc was used as an additive to generate the dimethyl malonate anion. The researchers also explored the use of corresponding *P*,*N*-type ligand (*S*,*S*,*Rp*)-**15b** under the same catalytic conditions, which resulted in competitive chemical yields but slightly decreased enantioselectivity in the chiral alkylation products.

### 3.2. Cu-Catalyzed [3 + 2] 1,3-Dipolar Cycloaddition

**Scheme 11:**
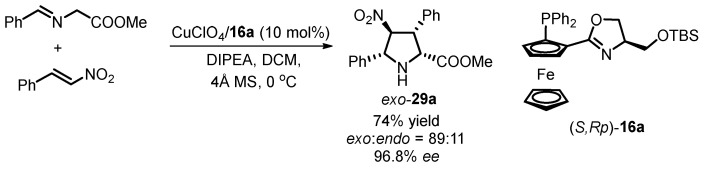
Cu(I)-catalyzed [3 + 2] 1,3-dipolar cycloaddition with nitroalkenes.

The *FOXAP* compounds have proven to be effective ligands in transition metal-catalyzed [3 + 2] 1,3-dipolar cycloaddition of azomethine ylides, facilitating the synthesis of enantioenriched pyrrolidines with high stereoselectivity [51,52,53,54,55,56,57,58]. Furthermore, recent investigations have explored the potential of siloxane-substituted oxazoline phosphine ferrocenes as alternative ligands in this cycloaddition. In a pioneering study by Zhou’s group in 2015, chiral siloxane-tagged ligands, exemplified by ligand (*S*,*Rp*)-**16a**, were evaluated as a candidate ligand for the Cu(I)-catalyzed cycloaddition of azomethine ylides with nitroalkenes [42]. The resulting cycloadduct *exo*-**29a** was obtained in 74% yield with an 89:11 d.r. and nearly 97% enantiomeric excess (Scheme 11).

**Scheme 12:**
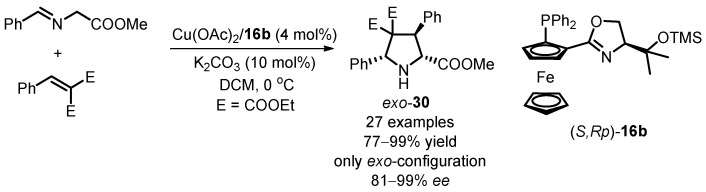
Cu(OAc)_2_-catalyzed [3 + 2] 1,3-dipolar cycloaddition with alkylidene malonates.

**Figure 3:**
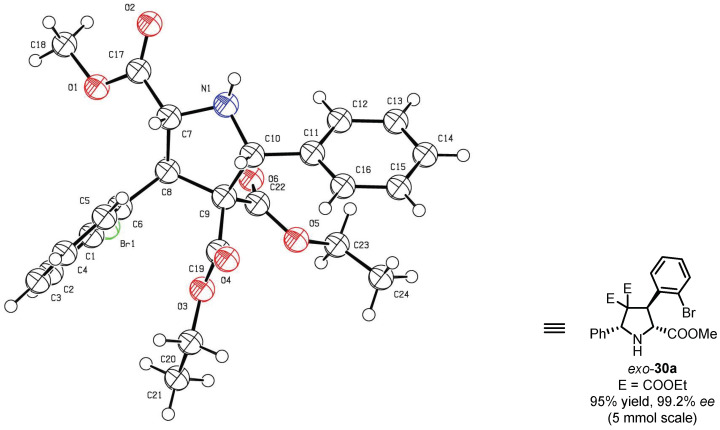
Catalytic result and X-ray structure of *exo*-**30a**. Copyright 2015 WILEY-VCH Verlag GmbH & Co. KGaA, Weinheim, Germany.

Zhou’s group proposed a practical strategy for synthesizing chiral *exo*-selective pyrrolidine-2,4,4-tricarboxylate derivatives through Cu(II)-catalyzed [3 + 2] 1,3-dipolar cycloaddition of azomethine ylides with alkylidene malonates in the presence of siloxane-substituted ligands (*S*,*Rp*)-**16** [47]. Optimizing the reaction conditions led to the model reaction providing the cyclization product with high yield and excellent enantioselectivity (up to 99% *ee*). The optimized conditions involved 4 mol% catalyst loading, 10 mol% K_2_CO_3_ as the additive base, and DCM as the solvent at 0 °C (Scheme 12). The study expanded the scope of reaction substrates, successfully preparing enantioselective pyrrolidines *exo*-**30** with different substituents, exhibiting enantioselectivity ranging from 78 to 99% *ee* under the optimized conditions. Importantly, the stable results obtained in the gram-scale preparation of *exo*-**30a** (Figure 3, 95% yield with 99.2% *ee*) indicated its potential industrial applicability as a chiral Cu complex system.

**Scheme 13:**
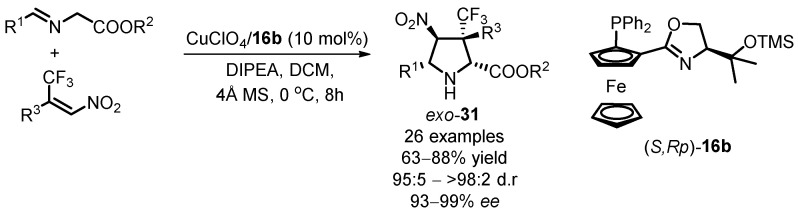
Cu(I)-catalyzed [3 + 2] 1,3-dipolar cycloaddition with *β*-trifluoromethyl nitroalkenes.

Chiral poly-substituted pyrrolidine derivatives containing a trifluoromethyl unit with a quaternary stereogenic center can be synthesized using similar catalytic systems [59]. The trifluoromethyl group is valuable in medicinal and agrochemistry due to its ability to modify the properties of target molecules. Zhou’s group used glycine imidate and *β*-trifluoromethyl nitroalkenes as substrates and observed that the target products, 4-nitro-3-(trifluoromethyl)-pyrrolidine-2-carboxylates *exo*-**31**, could be obtained by using the catalyst generated in situ from Cu(CH_3_CN)_4_ClO_4_ with the readily presented siloxane-substituted oxazoline phosphine ferrocene (*S*,*Rp*)-**16b** in DCM at 0 °C for 8 h (Scheme 13). The results of extended scope on reaction substrates indicate that chiral pyrrolidine compounds containing quaternary stereogenic centers can be synthesized with excellent stereoselectivity in most cases. The availability of easily accessible substrates and the potential bioactivity of enantioenriched trifluoromethylated pyrrolidine derivatives make this methodology particularly useful in medicinal and organofluorine chemistry.

**Scheme 14:**
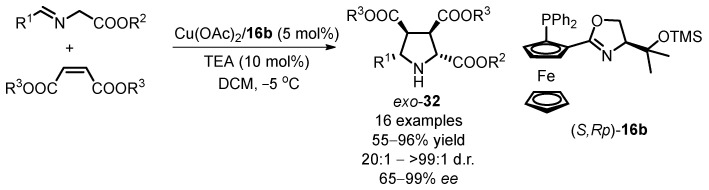
Cu(II)-catalyzed [3 + 2] 1,3-dipolar cycloaddition with maleate esters.

In a recent advancement, Zhou’s group presented an enhanced method for obtaining chiral pyrrolidine-2,3,4-tricarboxylates through enantioselective [3 + 2] 1,3-dipolar cycloaddition of azomethine ylides [60]. Initial screening involving diethyl fumarate as the dipolarophile yielded unsatisfactory results. The stereoselectivity of the target product, chiral pyrrolidine derivatives, was substantially improved by substituting diethyl maleates for diethyl fumarates. The Cu(II) complex, consisting of Cu(OAc)_2_ and the siloxane-substituted ligand (*S*,*Rp*)-**16b**, facilitated the generation of the desired *exo*-cycloadduct with excellent outcome (up to 96% yield, 99:1 d.r., and 96% *ee*). Further exploration of substrate reactions revealed that the position of the substituent on the phenyl group of the azomethine ylide significantly influenced both catalytic activity and stereoselectivity. The pyrrolidine-2,3,4-tricarboxylate compounds *exo*-**32** were obtained with the highest stereoselectivity of 99:1 d.r. and 99% *ee*, underscoring the success of this improved methodology (Scheme 14). This development not only enhances the efficiency of the enantioselective cycloaddition process but also provides valuable insights into the impact of substrate modifications on the reaction outcomes.

### 3.3. Cu-Catalyzed Self-Michael Reaction

**Scheme 15:**
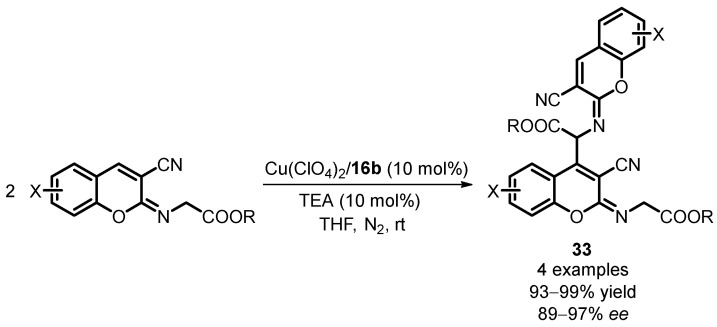
Asymmetric self-Michael addition of 2-[(3-cyano-2*H*-chromen-2-ylidene)amino]acetates.

Zhang reported an innovative synthesis of chiral dimeric chromene derivatives catalyzed by Cu(II) salts using the precursor 2-[(3-cyano-2*H*-chromen-2-ylidene)amino]acetates in a self-Michael addition reaction [61]. This method results in the formation of imino-chromene derivatives **33** with remarkable fluorescence properties, making them highly suitable for applications as optical whitening agents, laser dyes, and fluorescent probes in the medical field. Efforts to achieve enantioselective formation of the Michael addition with the siloxane-tagged FOXAP ligand (*S*,*Rp*)-**16b** led to the synthesis of 2-imino-chromene dimers with enantiomeric excess ranging from 89% to 97% (Scheme 15). This highlights the potential of the siloxane-substituted ligands in enabling the controlled and enantioselective synthesis of chiral chromene derivatives with desirable fluorescence properties.

### 3.4. Further Derivation of Siloxane-Substituted Oxazoline Ferrocenes

**Scheme 16:**
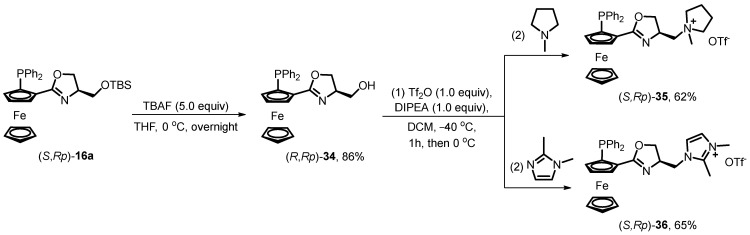
Synthetic schemes for establishing ionic-tagged oxazoline phosphine ferrocene ligands.

As discussed in Section 2.2.3, siloxane-substituted oxazoline phosphine ferrocene ligands can undergo a subsequent desilylation process (Scheme 16). The resulting hydroxyl group can be further functionalized with ionic groups, enhancing the solubility of chiral ionic-tagged ligands and their corresponding transition metal complexes in polar solvents, particularly ionic liquids. This advancement offers a sustainable approach for the recovery of expensive and environmentally unfriendly transition metal complexes in asymmetric catalytic reactions.

**Scheme 17:**
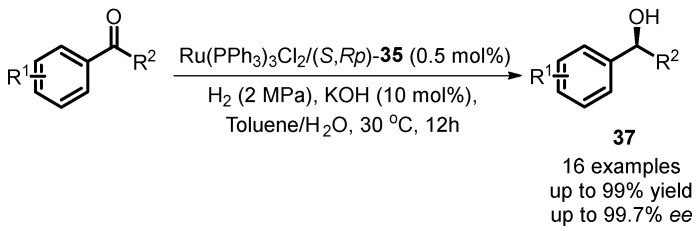
Asymmetric hydrogenation of aromatic ketones with quaternary ammonium tagged oxazoline phosphine ferrocene ligand.

In 2015, Zhang introduced an innovative recyclable ionic-tagged ferrocene-ruthenium catalyst system for the asymmetric hydrogenation of aromatic ketones (Scheme 17) [62]. The study revealed that the newly developed ferrocene-oxazoline phosphine ligand (*S*,*Rp*)-**35**, incorporating a quaternary ammonium ion group, exhibited outstanding catalytic performance in the hydrogenation reaction, yielding high product yields (up to 99%) and enantioselectivity (up to 99.7% *ee*).

Moreover, the research provided insights into the method for recycling ionic fragment-containing Ru complexes. The chosen ionic liquid, [Bmim]PF_6_, served as the immobilized phase for the chiral catalyst, facilitating the recovery of ionic-tagged transition metal complexes. Catalytic results demonstrated that the stereoselectivity of the chiral alcoholic product could be enhanced to 99.9% *ee* with the involvement of the ionic liquid. The asymmetric reduction of the catalyst remained effective for at least six runs with a minimum of 92.2% *ee* for product **37a** (Table 1). Mass spectrometry analysis of the catalyst anion after the sixth run confirmed the stability of the anionic group of chiral ligand. This approach highlights the potential for sustainable and efficient recycling in asymmetric catalytic processes.

**Scheme 18:**
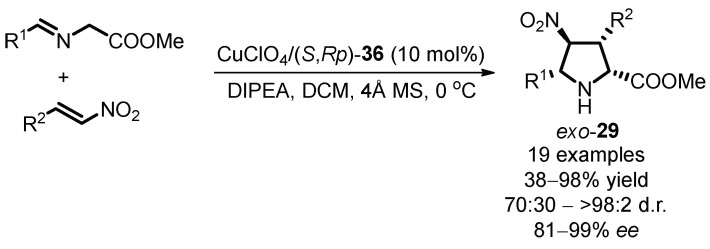
Cu(I)-catalyzed asymmetric [3 + 2] 1,3-dipolar cycloaddition with FimiOXAP ligands.

In the same year, Zhou introduced a scheme for synthesizing imidazolium-labelled oxazoline–phosphine–ferrocene ligands (*S*,*Rp*)-**36** (labeled as *FimiOXAP*) [42]. Commencing with siloxane-substituted oxazoline ferrocene (*S*,*Rp*)-**16a**, these imidazolium-tagged ligands were successfully obtained through a three-step sequential reaction. Meanwhile, five distinct ligands with varying anions were prepared by anion exchange. Subsequently, this class of chiral ligands found successful application in Cu(I)-catalyzed enantioselective [3 + 2] 1,3-dipolar cycloaddition of azomethine ylides with nitroalkenes (Scheme 18). In-depth studies into the reaction mechanism led to the conclusion that the electrostatic interaction of the charged ionic groups in the side chain of oxazoline can further enhance the stereoselectivity of the resulting optically active cycloadducts *exo*-**29**.

Remarkably, these ligands (*S*,*Rp*)-**36**, along with their corresponding transition metal complexes, exhibited excellent solubility in ionic liquids owing to the incorporation of ionophilic groups in the chiral ligand structure. Leveraging this unique property, the cycloaddition reaction smoothly progressed under the combined system of an ionic liquid and an organic solvent. Notably, the chiral complex catalyst displayed easy recyclability, maintaining robust conversion and enantioselectivity of *exo*-**29b** even after four consecutive runs (Table 2). This pioneering approach showcases the potential of ionic tagging for enhancing stereoselectivity and facilitating recyclability in asymmetric catalysis.

## 4. Summary and Outlook

In this brief review, we present the research achievements related to siloxane-substituted oxazoline ferrocene compounds in the past decade. The discussion on synthetic processes has unveiled several independent methodologies for crafting precursors of these compounds. Unlike conventional oxazoline ferrocene compounds, this section presents extended synthetic routes involving intricate substrates. Fortuitously, the effective synthetic strategies devised by researchers pave the way for their extensive derivatization and large-scale applications. Furthermore, the D*o*M procedure emerges as an indispensable pathway for obtaining highly stereoselective planar chiral ligands. The chemical inertness and substantial steric hindrance of the siloxane group empower the construction of *ortho*-functionalized ferrocene products with remarkable diastereoselectivity, even in the absence of additional additives. Siloxane-substituted oxazoline ferrocene ligands have transcended mono-phosphine ligands, venturing into the synthesis of unsymmetrical bisphosphine ligands and planar chiral *N*,*O*-catalysts.

On another front, siloxane-substituted oxazoline phosphine ferrocenes have proven to be highly effective ligands across mechanistically diverse transformations, yielding products with impressive stereoselectivity. In the realm of Cu-catalyzed [3 + 2] 1,3-dipolar cycloaddition of azomethine ylides, more than 70 chiral poly-substituted pyrrolidine derivatives have been synthesized with outstanding stereoselectivity. The consistent outcomes on a gram scale underscore the potential industrial applicability of Cu complex catalysts. Positive results in asymmetric allylic alkylation and Michael reactions further underscore the broad and practical applications of siloxane-substituted oxazoline phosphine ferrocenes.

A noteworthy development is the production of a series of ionic-tagged chiral ligands through further functionalization of the oxazoline side chain, implemented in recoverable catalytic systems. In the arena of asymmetric catalysis, these ionic-tagged catalysts exhibit comparable, if not superior, catalytic efficacy compared to traditional FOXAP ligands. The solubility of these ligands in ionic liquids facilitates the recovery of expensive and environmentally unfriendly transition metal complexes, marking a notable advancement in sustainable catalytic processes.

While the recent advancements in siloxane-substituted oxazoline ferrocene ligands have shown promise in transition metal-catalyzed asymmetric reactions, there remains substantial room for improvement and further exploration in this field. Addressing the following aspects could pave the way for enhanced applications and innovative developments:

1. *Optimizing Steric Hindrance*: Siloxane-substituted oxazoline ferrocene ligands, with their tunable side-chain structure, provide superior steric hindrance compared to conventional oxazoline ferrocene ligands. This characteristic is particularly valuable in the preparation of pharmaceutical fragments or natural products requiring precise steric hindrance. Future research could focus on exploiting this flexibility to achieve optimal stereoselective results in challenging synthesis processes.

2. *Functionalization and Solubility Enhancement*: The derivatization of chiral siloxane-substituted oxazoline ferrocene compounds opens avenues for further functionalization as chiral ligands and catalysts. Incorporating ionophilic or hydrophilic groups into the chiral side chain can enhance the solubility of these ligands and their corresponding transition metal complexes in ionic liquids or pure water. This advancement could lead to transition metal-catalyzed asymmetric reactions conducted in environmentally friendly solvents, aligning with green chemistry principles.

3. *Unique Catalytic Properties*: The electron-rich siloxane group’s ability to attract electrophilic reagents and repel nucleophiles presents an intriguing challenge and opportunity. Understanding and harnessing this unique property could offer valuable insights into optimizing reaction conditions and improving stereoselectivity. Further research is essential to unravel the implications of the siloxane-substituted oxazoline ferrocene’s distinctive catalytic behavior.

Our research group is actively exploring these subjects, aiming to contribute to the ongoing progress in the synthesis and application of siloxane-substituted oxazoline ferrocene compounds. As we delve deeper into these investigations, we anticipate uncovering new possibilities and refining existing methodologies for more sustainable and efficient asymmetric catalysis.

## Figures and Tables

**Scheme 6:**
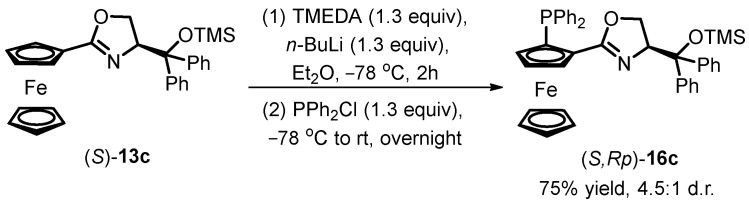
D*o*M procedure of oxazoline ferrocene with a bulky substituent.

**Table 1:** Selected example of recycled catalysts in asymmetric hydrogenation *^a^*.

Chiral Product	Run	Yield %	*ee* %
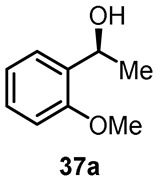	1	99	>99.9
	2	99	99.9
	3	97	97.8
	4	84	93.4
	5	73	92.5
	6	67	92.2

*^a^* catalytic reaction condition: substrate (1 mmol) concentration of 0.5 M in solvent and KOH (0.1 mmol) as base under 2 MPa of H_2_, 30 °C, 12 h, substrate/catalyst = 200:1.

**Table 2:** Selected example of catalyst recycling in asymmetric 1,3-dipolar cycloaddition *^a^*.

Chiral Product	Run	Yield %	d.r. (*exo*:*endo*)	*ee* % (*exo*)
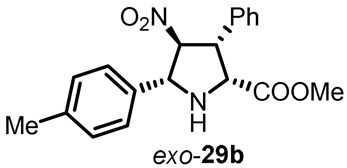	1	85	94:6	97.0
	2	71	85:15	92.6
	3	68	93:7	93.8
	4	74	82:18	91.4

*^a^* catalytic reaction condition: glycine imino ester (0.1 mmol), nitroalkene (0.11 mmol) in 1 mL of DCM/[Bmim]PF_6_ as solvent and DIPEA (0.01 mmol) as base, 0 °C, 12 h, substrate/catalyst = 10:1. Chiral ligand = (*S*,*Rp*)-**36**.

## List of Abbreviations

BSA	*N*,*O*-bis(trimethlysilyl)acetamide
d.r.	diastereoselectivity ratio
DAST	diethylaminosulphur trifluoride
DCM	dichloromethane
DIPEA	*N*,*N*-diisopropylethylamine
DMAP	4-(dimethylamino)pyridine
DMF	*N*,*N*-dimethylformamide
*ee*	enantiomeric excess
FOXAP	oxazoline phosphine ferrocene (ligands)
IL	ionic liquids
*o*-Tol	2-methylphenyl
PEG	polyethylene glycol
rt	room temperature
TBAF	tetrabutylammonium fluoride
TBS	*tert*-butyldimethylsilyl
TEA	triethylamine
THF	tetrahydrofuran
TMEDA	*N*,*N*,*N*′,*N*′-tetramethylethylenediamine
TMS	trimethylsilyl
TsCl	tosyl chloride
